# Selective Homocysteine Lowering Gene Transfer Improves Infarct Healing, Attenuates Remodelling, and Enhances Diastolic Function after Myocardial Infarction in Mice

**DOI:** 10.1371/journal.pone.0063710

**Published:** 2013-05-13

**Authors:** Ilayaraja Muthuramu, Frank Jacobs, Neha Singh, Stephanie C. Gordts, Bart De Geest

**Affiliations:** Centre for Molecular and Vascular Biology, Catholic University of Leuven, Leuven, Belgium; University of Buenos Aires, Faculty of Medicine. Cardiovascular Pathophysiology Institute., Argentina

## Abstract

**Background and aims:**

Homocysteine levels predict heart failure incidence in prospective epidemiological studies and correlate with severity of heart failure in cross-sectional surveys. The objective of this study was to evaluate whether a selective homocysteine lowering intervention beneficially affects cardiac remodelling and cardiac function after myocardial infarction (MI) in a murine model of combined hypercholesterolemia and hyperhomocysteinemia.

**Methodology and principal findings:**

A selective homocysteine lowering gene transfer strategy was evaluated in female C57BL/6 *low density lipoprotein receptor* (*Ldlr*)^−/−^ *cystathionine-ß-synthase* (*Cbs*)^+/−^ deficient mice fed a hyperhomocysteinemic and high saturated fat/high cholesterol diet using an E1E3E4-deleted hepatocyte-specific adenoviral vector expressing *Cbs* (AdCBS). MI was induced by permanent ligation of the left anterior descending coronary artery 14 days after saline injection or gene transfer. AdCBS gene transfer resulted in a persistent more than 5-fold (p<0.01) decrease of plasma homocysteine levels and significantly improved endothelial progenitor cell function. Selective homocysteine lowering enhanced infarct healing as indicated by a 21% (p<0.01) reduction of infarct length at day 28 after MI and by an increased number of capillaries and increased collagen content in the infarct zone. Adverse remodelling was attenuated in AdCBS MI mice as evidenced by a 29% (p<0.05) reduction of left ventricular cavity area at day 28, by an increased capillary density in the remote myocardium, and by reduced interstitial collagen. The peak rate of isovolumetric relaxation was increased by 19% (p<0.05) and the time constant of left ventricular relaxation was reduced by 21% (p<0.05) in AdCBS MI mice compared to control MI mice, indicating improved diastolic function.

**Conclusion/significance:**

Selective homocysteine lowering gene transfer improves infarct healing, attenuates remodelling, and significantly enhances diastolic function post-MI in female C57BL/6 *Ldlr* ^−/−^ *Cbs* ^+/−^ mice. The current study corroborates the view that hyperhomocysteinemia exerts direct effects on the myocardium and may potentiate the development of heart failure.

## Introduction

Epidemiological studies have demonstrated an association between plasma homocysteine levels and left ventricular mass [1] and function [2] in asymptomatic subjects. Furthermore, homocysteine levels predict heart failure incidence in prospective studies and correlate with severity of heart failure in cross-sectional surveys [3], [4], [5]. The strong relationship between plasma homocysteine levels and heart failure incidence and severity may reflect cause or confounding. A causal role of homocysteine in cardiac dysfunction is suggested by experimental animal and *in vitro* studies demonstrating direct effects of homocysteine on cardiomyocytes [5]. Hyperhomocysteinemia affects myocardial metabolism [6] and can induce cardiomyocyte dysfunction and apoptosis. Hyperhomocysteinemia may promote myocardial dysfunction via increased oxidative stress [7] and by promoting myocardial fibrosis [8]. In a rat model of hyperhomocysteinemia induced by chronic methionine administration, interstitial myocardial fibrosis was associated with increased expression of transforming growth factor-ß1 and of tissue inhibitor of metalloproteinase-2 and with increased JNK activation [9]. Mild hyperhomocysteinemia in heterozygous *cystathionine-β-synthase* (*Cbs)* deficient mice was accompanied by cardiomyocyte hypertrophy and myocardial collagen accumulation [10], which is consistent with similar observations in normotensive and hypertensive rats with diet-induced hyperhomocysteinemia [8], [11]. However, most prior experimental animal studies on the effects of homocysteine on the myocardium involved different diets in the control group and intervention group. Therefore, the outcome in these studies may be affected by dietary effects unrelated to homocysteine levels.

Hypercholesterolemia may also have direct adverse effect on the myocardium and on cardiac remodelling post-myocardial infarction (MI). In Framingham Heart Study participants free of coronary heart disease at baseline, high non-high density lipoprotein (HDL) cholesterol levels were independently associated with heart failure incidence after adjustment for interim myocardial infarction and clinical covariates [12]. Furthermore, in patients with a first myocardial infarction, hypercholesterolemia is associated with a more pronounced deterioration of the left ventricular function [13]. In agreement, hypercholesterolemia in C57BL/6 *low density lipoprotein receptor* (*Ldlr*)^−/−^ mice results in more pronounced adverse ventricular remodelling and in a worse cardiac function after permanent ligation of the left anterior descending coronary artery (LAD) [14].

The objective of this study was to evaluate whether a selective homocysteine lowering intervention beneficially affects cardiac remodelling and cardiac function after MI in a murine model of combined hypercholesterolemia and hyperhomocysteinemia. Therefore, a selective homocysteine lowering gene transfer strategy without any dietary changes during the intervention phase was evaluated in C57BL/6 *low Ldlr* ^−/−^ *Cbs* ^+/−^ mice fed a hyperhomocysteinemic and high saturated fat/high cholesterol diet. Our hypothesis was that selective homocysteine lowering gene transfer would attenuate cardiac remodelling and would improve cardiac function after permanent ligation of the LAD.

## Materials and Methods

### Ethics Statement

All experimental procedures in animals were performed in accordance with protocols approved by the Institutional Animal Care and Research Advisory Committee of the Catholic University of Leuven.

### Construction, Generation, and Production of E1E3E4-deleted Adenoviral Gene Transfer Vectors

The construction of the E1E3E4-deleted adenoviral vector AdCBS, which induces hepatocyte-specific expression of cystathionine-β-synthase (CBS), has been described previously [15]. This vector contains the 1.2 kb DC172 promoter [16], consisting of an 890 bp human *α_1_-antitrypsin* promoter and two copies of the 160 bp α_1_-*microglobulin* enhancer, upstream of the 5′ untranslated region of the human *apo A-I* gene that contains the first intron, the 1.7 kb cDNA sequence of murine *Cbs,* and 2 copies of the 774 bp *hepatic control region-1*. The E1E3E4-deleted control vector Adnull does not contain an expression cassette [17]. Large scale vector production was performed as described previously [17].

### In vivo Experiments

To induce hypercholesterolemia and hyperhomocysteinemia, female C57BL/6 *Ldlr* ^−/−^ *Cbs* ^+/−^ mice [15] were fed a folate-depleted, methionine-enriched diet (TD00205; 0.2 mg/kg folic acid, 4.1 g/kg L-methionine; Harlan Teklad, Horst, The Netherlands) supplemented with 0.2% (w/w) cholesterol and 10% (v/w) coconut oil *ad libitum*, starting from the age of 12 weeks. Three weeks after initiation of the diet, mice were injected intravenously with 5×10^10^ adenoviral particles of AdCBS via the tail vein. Control mice were injected with the same dose of Adnull (n = 7) or with saline buffer (n = 6). The equivalency of Adnull and saline controls with regard to different outcome measures has been demonstrated in several studies [14], [18], [19], which indicates that the E1E3E4-deleted adenoviral vectors *per se* do not affect results in one or another direction. Therefore and since no difference or trend for difference was observed between the Adnull and saline injected mice for all parameters investigated in the current study, data of both control groups were consistently pooled. The experimental diet was maintained throughout the entire duration of the experiments. Three different reference groups were included in the study: sham C57BL/6 *Ldlr* ^−/−^ *Cbs* ^+/−^ mice and sham AdCBS C57BL/6 *Ldlr* ^−/−^ *Cbs* ^+/−^ mice fed the same diet as the MI groups, and sham C57BL/6 mice fed normal chow.

### Determination of Lipoprotein Cholesterol Levels

Mouse lipoproteins were separated by density gradient ultracentrifugation in a swing-out rotor as described before [20]. Fractions were stored at −20°C until analysis. Total cholesterol in plasma and lipoprotein fractions was determined with commercially available enzymes (Roche Diagnostics, Basel, Switzerland). Precipath L (Roche Diagnostics) was used as a standard.

### Determination of Homocysteine Levels in Murine Plasma Samples

Total plasma homocysteine (tHcy), expressed as the total level of homocysteine after quantitative reductive cleavage of all disulfide bonds, was measured with an automated fluorescence polarization immunoassay using an Abbott IMX immunoanalyzer (Abbott Diagnostics, Abbott Park, IL, USA) [15]. Blood was collected from the retro-orbital plexus and anticoagulated with ethylenediaminetetraacetic acid (EDTA; final concentration 5 mM). Samples were kept on ice and plasma was obtained following centrifugation at 1100 g for 10 minutes. Plasma samples were kept at −20°C until analysis.

### Murine Endothelial Progenitor Cell (EPC) Culture Assay

Spleen mononuclear cells were cultivated and EPCs were quantified as described before [21]. Spleen mononuclear cells were isolated 14 days after gene transfer by Ficoll-based centrifugation and seeded onto fibronectin (40 µg/ml)-coated 24-well plates (Sigma, Steinheim, Germany) at a density of 6×10^6^ cells/well in 0.5 ml EGM-2MV BulletKit medium (Cambrex, East Rutherford, NJ, U.S.A.) according to the instructions of the manufacturer. After 7 days of culture, medium was removed and adhered cells were stained for DiI-acLDL (Invitrogen, Carlsbad, CA, U.S.A.) (6.6 µg/ml) for 4 hours and then FITC-labeled isolectin (Invitrogen) (10 µg/ml) for 1 hour. The number of EPCs, identified as DiI-acLDL isolectin double positive cells, per microscopy field was quantified.

### EPC Migration Assay

EPC migration was studied by using modified Boyden chambers (Costar, Avon, France) as described [22]. After 7 days of culture, spleen EPCs were seeded in the upper chamber with a density of 2×10^4^ cells per well in 200 µl EGM-2MV medium. In selected experiments, stromal derived factor-1α (SDF-1α) (100 ng/ml; R&D Systems, Minneapolis, MN, USA) was added in the lower chamber. EPCs were allowed to migrate for 5 hours at 37°C. For quantification, cell nuclei were stained with 4′,6-diamidine-2-phenylidole dihydrochloride (DAPI; Invitrogen) and EPCs migrated into the lower chamber were counted manually in randomly selected microscopy fields.

### Quantification of CBS Expression in the Liver

Livers were homogenized in lysis buffer (20 mM HEPES pH 7.2, 5 mM KCl, 5 mM MgCl_2_, 0.5% TRITON X-100) in the presence of a complete protease inhibitor (Roche). Next, homogenates were centrifuged for 10 minutes at 13000 g and 4°C and 50 µg of protein were separated on a 10% polyacrylamide gel. Proteins were transferred to a nitrocellulose membrane by wet blotting in transfer buffer (25 mM Tris, 190 mM glycine, 20% (v/v) methanol, 0.1% SDS, pH 7.5) for 2 hours at a constant current of 180 mA. Following overnight incubation with a 1∶1000 dilution of goat anti-mouse CBS antibody (sc-46830; Santa Cruz Biotechnology Inc., Santa Cruz, CA, U.S.A.) and subsequent incubation with horseradish peroxidase-conjugated rabbit anti-goat antibody (DAKO, Glostrup, Denmark) in a 1∶1000 dilution, the membrane was developed using ECL detection reagent (Amersham Biosciences). Films were scanned and CBS levels (molecular weights 63 kDa) were quantified using ImageJ software (Wayne Rasband, National Institutes of Health, USA).

### Myocardial Infarction

Two weeks after gene transfer or saline injection, myocardial infarction (MI) was induced in female C57BL/6 *Ldlr* ^−/−^ *Cbs* ^+/−^ mice by permanent ligation of the left anterior descending coronary artery (LAD) as described [14].

### In vivo Hemodynamic Measurements

Invasive hemodynamic measurements were performed 28 days after MI. Mice were anesthetized by intraperitoneal administration of 1.4 g/kg urethane (Sigma, Steinheim, Germany). Body temperature was maintained with a heating pad and monitored with a rectal probe. An incision in the right carotid artery was made with a 26-gauge needle between a distal and proximal non-occlusive ligation of the artery. A 1.1 French Millar pressure catheter (SPR-67/NR; Millar instruments, Houston, Texas, USA) was inserted and advanced to the left ventricle (LV). After stabilization of the catheter, heart rate, maximal systolic LV pressure, minimal diastolic LV pressure, the peak rate of isovolumetric LV contraction (dP/dt_max_), and the peak rate of isovolumetric LV relaxation (dP/dt_min_) were measured. The end-diastolic LV pressure was calculated manually from the pressure in function of time curves. The time constant of isovolumetric LV pressure fall (tau) was calculated using the method of Weiss *et al.* [23]. Arterial blood pressure measurements were obtained after withdrawal of the catheter from the LV to the ascending aorta. Data were registered with Powerlab Bridge Amplifier and Chart Software (sampling rate 2000 Hz; Fysicon, Oss, the Netherlands).

### Tissue Preparation for Histological and Morphometric Analysis

Mice were perfused via the abdominal aorta with phosphate-buffered saline (PBS) and hearts were arrested in diastole by CdCl (100 µl; 0.1 N), followed by perfusion fixation with 1% paraformaldehyde in PBS. Hearts and lungs were dissected and weighed. Hearts were post-fixated overnight in 1% paraformaldehyde, embedded in paraffin, and 6 µm thick cross-sections at 130 µm spaced intervals were made extending from the apex to the basal part of the left ventricle.

### Morphometric Analysis of Left Ventricle Remodelling

LV remodelling was assessed by morphometric analysis on mosaic images of Sirius red-stained heart cross-sections using Axiovision 4.6 software (Zeiss, Zaventem, Belgium). Infarct size (%) at day 28 post-MI was calculated according to Takagawa *et al.* [24] by dividing the sum of midline infarct lengths from all sections by the sum of midline LV circumferences from all sections and multiplying by 100. Midline infarct length was defined as the midline length of infarct that included more than 50% of the whole thickness of the myocardial wall. Whole LV area (µm^2^), LV cavity area (µm^2^), LV remote muscle area (µm^2^; including the septum), and infarct area (µm^2^) were analyzed. Infarct wall thickness (µm) was measured at equidistant points over the infarct area perpendicular to the infarcted wall. All geometric measurements were computed in a blinded fashion from representative tissue sections of 4 separate regions and the average value was used to represent that animal for statistical purposes.

### Analysis of Collagen Deposition

To measure collagen content in the infarct and in the interstitium, Sirius Red staining was performed as previously described [25]. Sirius Red polarization microscopy on a Leica RBE microscope with KS300 software (Zeiss) was used to quantify thick closely packed mature collagen fibers as orange-red birefringent and loosely packed less cross-linked and immature collagen fibers as yellow-green birefringent. Collagen positive area was normalized to the total LV remote area or infarct area and was expressed as percentage. Any perivascular fibrosis was excluded from the analysis of interstitial collagen. Perivascular fibrosis was quantified as the ratio of the fibrosis area surrounding the vessel to the total vessel area. Two mid-ventricular sections were studied per animal.

### Immunohistochemistry

Cardiomyocyte hypertrophy was analyzed on paraffin sections stained with rabbit anti-mouse laminin (Sigma; 1/50) by measuring the cardiomyocyte cross-sectional area (µm^2^) of at least 200 randomly selected cardiomyocytes in the non-infarcted LV myocardium. Two mid-ventricular cross-sections were analyzed per mouse. Cardiomyocyte density was determined on the same laminin stained sections by counting the number of cross-sectioned round shaped cardiomyocytes per mm^2^ of cardiomyocyte-covered LV myocardium.

Capillary density in the infarct area, the infarct border zone, and the non-infarcted myocardium was determined on CD31 stained sections using rat anti-mouse CD31 antibodies (BD; 1/500). Relative vascularity in the non-infarcted myocardium was determined as ^hello^(capillary density (number/mm^2^)/cardiomyocyte density (number/mm^2^))/cardiomyocyte cross-sectional area (µm^2^)] [26].

### Statistical Analysis

All data are expressed as means ± standard error of the means (S.E.M.). Longitudinal homocysteine and cholesterol data were compared between day 0 and later time-points by Kruskal-Wallis test followed by Dunn multiple comparison posttest using Instat3 (GraphPad Software, San Diego, CA, USA). Infarct parameters were generally compared with an unpaired Student’s t-test. When indicated, a logarithmic transformation, a square root transformation, or a non-parametric Mann-Whitney test was performed. Parameters between three groups were compared by one-way analysis of variance followed by Tukey multiple comparison post-test using GraphPad Instat. When indicated, a logarithmic transformation was performed or a non-parametric test Kruskal-Wallis Test followed by Dunn multiple comparison post-test was applied. A two-sided p-value of less than 0.05 was considered statistically significant.

## Results

### AdCBS Gene Transfer Selectively Lowers Homocysteine Levels in C57BL/6 *Ldlr^−/−^ Cbs^+/−^* Mice

**Figure 1:**
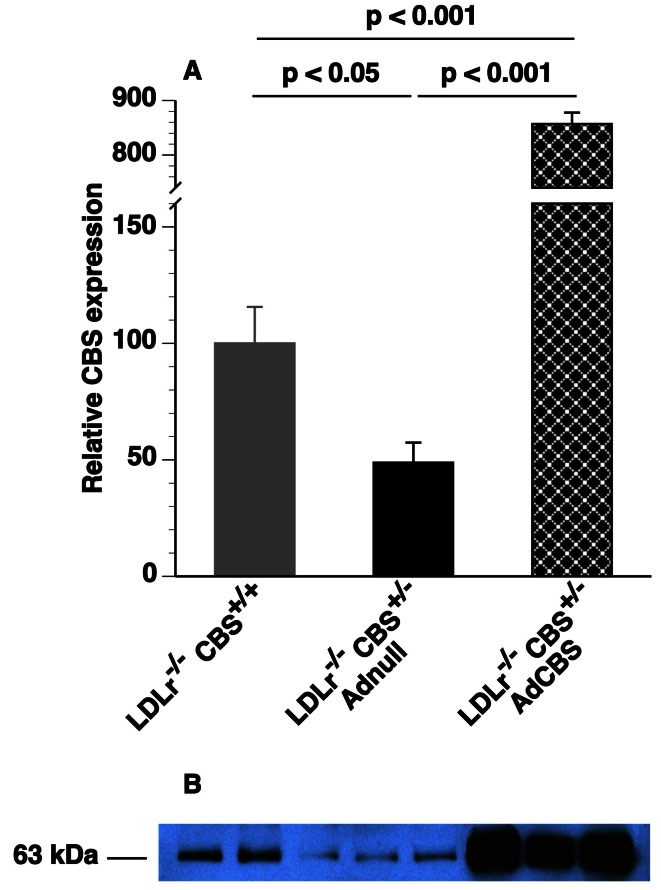
(A) Relative protein expression levels of CBS in the liver of C57BL/6 *Ldlr^−/−^ Cbs^+/+^* mice (grey bar) and of C57BL/6 *Ldlr^−/−^ Cbs^+/−^* mice 2 weeks after gene transfer with 10^11^ particles of Adnull (black bar) or 10^11^ particles of AdCBS (black crosshatched bar). Data in Adnull- and AdCBS-treated mice were calculated using values in C57BL/6 *Ldlr^−/−^ Cbs^+/+^* mice as the denominator. All data are shown as means ± SEM (n = 7 to 9 for each condition). (B) Representative Western blot of CBS expression in the liver of C57BL/6 *Ldlr^−/−^ Cbs^+/+^* mice (lanes 1 and 2) and of C57BL/6 *Ldlr^−/−^ Cbs^+/−^* mice injected with 10^11^ particles of Adnull (lanes 3–5) or the same dose of AdCBS (lanes 6–8).

In pilot experiments, overexpression of murine CBS in the liver after AdCBS gene transfer in C57BL/6 *Ldlr* ^−/−^ *Cbs* ^+/−^ mice was confirmed by Western blot (Figure 1).

**Figure 2:**
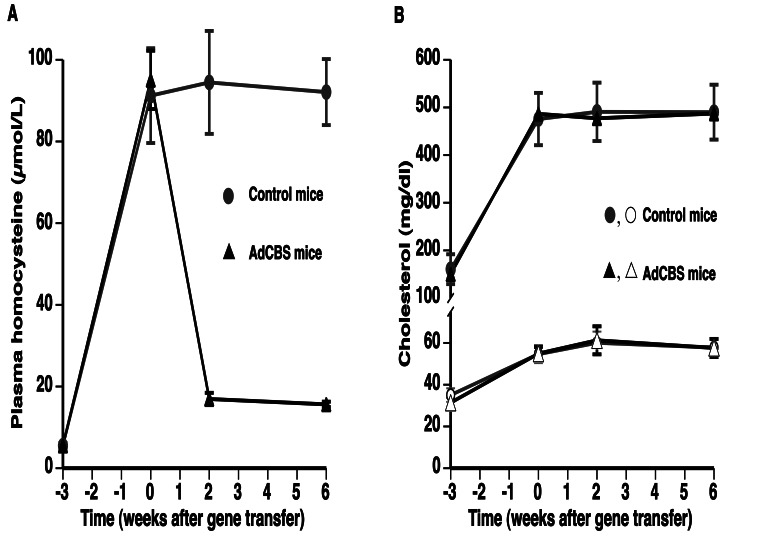
Time course of plasma homocysteine levels (A) and plasma cholesterol (closed symbols) and HDL cholesterol levels (open symbols) (B) in female C57BL/6 *Ldlr* ^−/−^ *Cbs* ^+/−^ control mice or in female C57BL/6 *Ldlr* ^−/−^ *Cbs* ^+/−^ mice injected with 5×10^10^ particles of AdCBS. A hyperhomocysteinemic and high saturated fat/high cholesterol diet (0.2 mg/kg folic acid, 4.1 g/kg L-methionine, 1.25% cholesterol (w/w), and 10% coconut oil (v/w)) was initiated 3 weeks before adenoviral gene transfer or saline injection and maintained throughout the experiment. The 0 week time point corresponds to the time point of gene transfer in the intervention group. Data are expressed as means ± S.E.M. (n = 8).

A folate-depleted, methionine-enriched diet supplemented with cholesterol and coconut oil was initiated in female C57BL/6 *Ldlr* ^−/−^ *Cbs* ^+/−^ mice at the age of 12 weeks to induce hyperhomocysteinemia and hypercholesterolemia. Three weeks later, gene transfer was performed with 5×10^10^ particles of AdCBS to lower homocysteine levels. Compared to plasma homocysteine levels at the time of gene transfer (95.1±7.1 µM), AdCBS gene transfer resulted in a 5.6-fold (p<0.01) and a 6.1-fold (p<0.01) decrease of plasma homocysteine concentrations at day 14 and at day 42 after gene transfer, respectively (Figure 2A). No alterations of plasma homocysteine levels were observed in control mice injected with saline buffer or Adnull control vector. Figure 2B illustrates that plasma cholesterol levels and HDL cholesterol levels were stable in the final 6 weeks of the experiment in both groups. In addition, VLDL, IDL, and LDL cholesterol levels were stable from 0 till 6 weeks (data not shown).

### Selective Homocysteine Lowering Improves Infarct Healing and Attenuates Left Ventricular Remodelling after Myocardial Infarction

Myocardial infarction (MI) was induced by permanent ligation of the left anterior descending coronary artery 2 weeks after gene transfer or saline injection. Experimental mortality was low: one dead mouse in each MI group. Heart and lung weights at day 28 after MI in comparison with sham C57BL/6 *Ldlr* ^−/−^ *Cbs* ^+/−^ mice fed the same diet are shown in Table 1. Heart weight was reduced by 28% (p<0.05) in AdCBS MI mice compared to control MI mice, which corresponds to the smaller volume of the former. Lung weight was 29% (p<0.05) lower in AdCBS MI mice than in control MI mice, which likely reflects pulmonary congestion in the latter. Similar data were obtained when heart and lung weights were normalized to tibia length (Table 1). To exclude major cardiac abnormalities induced by metabolic alterations in the sham C57BL/6 *Ldlr* ^−/−^ *Cbs* ^+/−^ mice, parameters in these mice were compared with sham C57BL/6 mice fed normal chow and with sham AdCBS treated C57BL/6 *Ldlr* ^−/−^ *Cbs* ^+/−^ mice fed the same diet as the control sham C57BL/6 *Ldlr* ^−/−^ *Cbs* ^+/−^ mice and both MI groups. Heart and lung weights were similar in the three different sham groups (Table 2).

**Table 1:** Heart and lung weights in sham C57BL/6 *Ldlr* ^−/−^ *Cbs* ^+/−^ mice and at 28 days after myocardial infarction in control and AdCBS treated C57BL/6 *Ldlr* ^−/−^ *Cbs* ^+/−^ mice.

	Sham	Control MI	AdCBS MI
Heart weight (mg)	142±10	279±27§§§	218±8§§§ *
Heart weight/tibia length (mg/mm)	8.59±0.53	16.4±1.7§§§	12.7±0.5§§§ *
Lung weight (mg)	163±7	228±20§	177±10*
Lung weight/tibia length (mg/mm)	9.94±0.41	13.5±1.3§	10.3±0.6*

Data are expressed as means ± S.E.M. (n = 11 for Control reference, n = 13 for Control MI, n = 25 for AdCBS MI).

§ p<0.05;

§§§ p<0.001 versus Sham.

* p<0.05 versus Control MI.

**Table 2:** Heart and lung weights of sham C57BL/6 mice and of sham C57BL/6 *Ldlr* ^−/−^ *Cbs* ^+/−^ and sham AdCBS treated C57BL/6 *Ldlr* ^−/−^ *Cbs* ^+/−^ mice.

	Sham C57BL/6	Sham C57BL/6*Ldlr* ^−/−^ *Cbs* ^+/−^	Sham AdCBS treated C57BL/6 *Ldlr* ^−/−^ *Cbs* ^+/−^
Heart weight (mg)	139±6	142±10	139±3
Heart weight/tibia length (mg/mm)	8.46±0.33	8.59±0.53	8.42±0.31
Lung weight (mg)	149±8	163±7	151±5
Lung weight/tibia length (mg/mm)	9.08±0.42	9.94±0.41	9.19±0.38

Sham C57BL/6 mice (n = 10) were fed normal chow. Sham C57BL/6 *Ldlr* ^−/−^ *Cbs* ^+/−^ (n = 11) and sham AdCBS treated (n = 11) C57BL/6 *Ldlr* ^−/−^ *Cbs* ^+/−^ mice were fed with folate-depleted, methionine-enriched diet supplemented with 0.2% cholesterol and 10% coconut oil. Data are expressed as means ± S.E.M.

**Figure 3:**
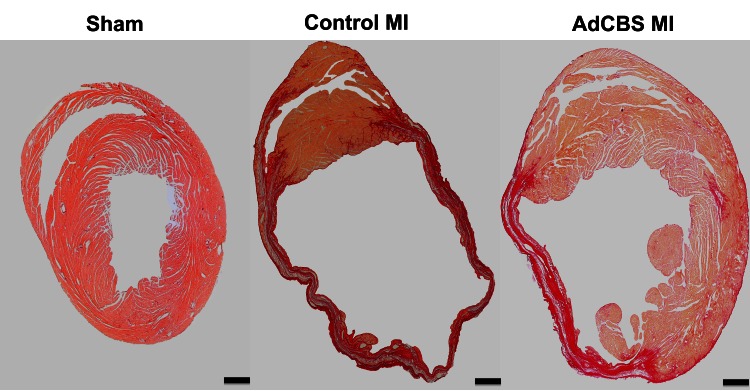
Representative Sirius red stained cross-sections of sham C57BL/6 *Ldlr* ^−/−^ *Cbs* ^+/−^ mice, control MI mice, and AdCBS MI mice at day 28 after ligation of the LAD. Morphometric analysis was performed on tissue sections of 4 separate regions using Axiovision 4.6 software (Zeiss). Scale bar represents 1 mm.

**Figure 4:**
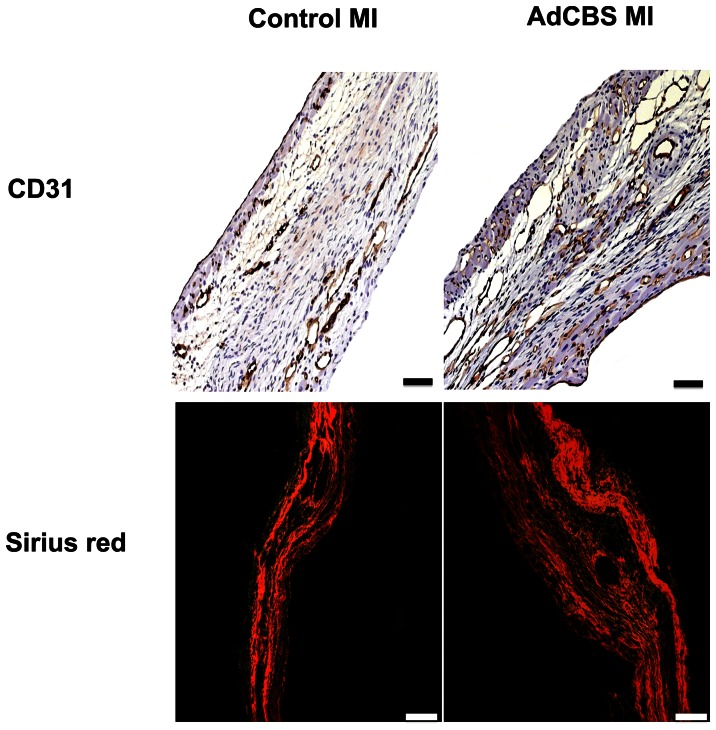
(Immuno)histochemical analysis of the infarct area in control MI and AdCBS MI mice at day 28 after ligation of the LAD. Representative photomicrographs show CD31 positive capillaries and Sirius red stained collagen viewed under polarized light. Scale bar represents 50 µm.

**Figure 5:**
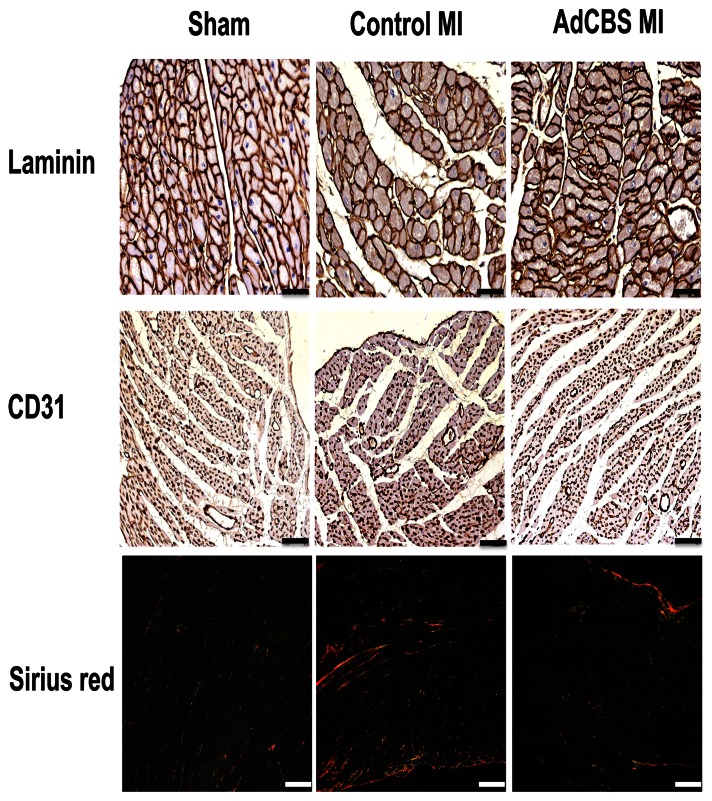
(Immuno)histochemical analysis of the remote myocardium of sham C57BL/6 *Ldlr* ^−/−^ *Cbs* ^+/−^ mice, control MI mice, and AdCBS MI mice 28 days after ligation of the LAD. Representative photomicrographs show laminin stained cardiomyocytes, CD31 positive capillaries, and Sirius red stained interstitial collagen viewed under polarized light. Scale bar represents 50 µm.

Morphometric data at day 28 after MI in comparison with sham C57BL/6 *Ldlr* ^−/−^ *Cbs* ^+/−^ mice fed the same diet are summarized in Table 3. A morphometric comparison of the three sham groups is shown in Table 4. Representative Sirius red stained cross-sections of sham C57BL/6 *Ldlr* ^−/−^ *Cbs* ^+/−^ mice, control MI mice, and AdCBS MI mice at day 28 after ligation of the LAD are shown in Figure 3. Infarct size at day 28 after MI, expressed as percentage of the left ventricular circumference, was significantly reduced in AdCBS MI mice compared to control MI mice (Table 3). This difference in infarct size reflected a 21% (p<0.01) decrease of absolute infarct length, suggesting improved infarct healing. Histological analysis of the infarct zone (Figure 4) was consistent with improved infarct healing as evidenced by a 22% (p = NS) and a 1.27-fold (p<0.05) increase of CD31 positive capillaries and of collagen content percentage, respectively, in the infarct zone (Table 5). Increased collagen content in the infarct zone induced by homocysteine lowering was predominantly the result of a higher mature collagen content in AdCBS MI mice (37.3±1.9% versus 27.3±2.7%; p<0.01). Improved infarct healing was associated with reduced expansive remodelling as evidenced by a 29% (p<0.05) reduction of left ventricular cavity area in AdCBS MI mice compared to control MI mice (Table 3). No significant differences were observed in the septal wall thickness and the left ventricular remote muscle area between AdCBS MI and control MI mice (Table 3). Therefore, the reduced heart weight in AdCBS MI mice (Table 1) reflects attenuation of left ventricular dilatation. Histological analysis of the remote myocardium (Figure 5) demonstrated similar cardiomyocyte cross-sectional area and cardiomyocyte density between both groups, but compared to sham mice significant cardiomyocyte hypertrophy was observed in both MI groups (Table 5). Capillary density in the remote myocardium was 25% (p<0.05) higher in the AdCBS MI group compared to the control MI group. Total interstitial collagen content was significantly lower in AdCBS MI mice than in control MI mice (Table 5). This difference in total interstitial collagen content was also reflected by a lower level of mature interstitial collagen in AdCBS MI mice (4.69±0.91 versus 7.20±1.50%; p = 0.069). Perivascular fibrosis was similar in both MI groups, but was significantly increased compared to sham mice (Table 5). Histological parameters in the three sham groups are shown in Table 6. Perivascular fibrosis was increased by 33% (p<0.001) in sham C57BL/6 *Ldlr* ^−/−^ *Cbs* ^+/−^ mice compared to chow-fed C57BL/6 mice (Table 6).

**Table 3:** Morphometric parameters of the left ventricle of Sham C57BL/6 *Ldlr* ^−/−^ *Cbs* ^+/−^ mice and morphometric analysis of the infarct and of left ventricular remodelling at 28 days after myocardial infarction in control and AdCBS treated C57BL/6 *Ldlr* ^−/−^ *Cbs* ^+/−^ mice.

	Sham	Control MI	AdCBS MI
Infarct length (µm)	N.A.	8590±610	6820±250**
Infarct size (% of circumference)	N.A.	60.3±3.0	53.7±1.4*
Infarct area (mm^2^)	N.A.	2.69±0.25	2.30±0.11
Infarct thickness (µm)	N.A.	351±20	363±7
Septal wall thickness (µm)	1140±40	1020±35	1120±36
LV remote muscle area(mm^2^)	9.27±0.47	6.59±0.41§§§	7.20±0.28§§§
LV cavity area (mm^2^)	3.94±0.33	11.9±0.6§§§	9.24±0.50§§§ *
Whole LV area (µm^2^)	13.2±0.5	21.2±0.8§§§	18.7±0.5§§§ *

Data are expressed as means ± S.E.M. (n = 11 for Sham, n = 13 for Control MI, n = 25 for AdCBS MI).

N.A.: not applicable.

§§§ p<0.001 versus Sham.

* p<0.05,

** p<0.01 versus Control MI. LV: left ventricular.

**Table 4:** Morphometric analysis of the left ventricle of sham C57BL/6 mice and of sham C57BL/6 *Ldlr* ^−/−^ *Cbs* ^+/−^ and sham AdCBS treated C57BL/6 *Ldlr* ^−/−^ *Cbs* ^+/−^ mice.

	Sham C57BL/6	Sham C57BL/6*Ldlr* ^−/−^ *Cbs* ^+/−^	Sham AdCBS treated C57BL/6 *Ldlr* ^−/−^ *Cbs* ^+/−^
LV cavity area (mm^2^)	3.88±0.44	3.94±0.33	3.65±0.28
LV remote muscle area (mm^2^)	9.65±0.45	9.27±0.47	9.10±0.16
Septal wall thickness (µm)	1090±30	1140±40	1070±20
Whole LV area (µm^2^)	13.5±0.6	13.2±0.5	12.8±0.3

Sham C57BL/6 mice (n = 10) were fed normal chow. Sham C57BL/6 *Ldlr* ^−/−^ *Cbs* ^+/−^ (n = 11) and sham AdCBS treated (n = 11) C57BL/6 *Ldlr* ^−/−^ *Cbs* ^+/−^ mice were fed with folate-depleted, methionine-enriched diet supplemented with 0.2% cholesterol and 10% coconut oil. Data are expressed as means ± S.E.M.

LV: left ventricular.

**Table 5:** Histological parameters of the left ventricular myocardium of Sham C57BL/6 *Ldlr* ^−/−^ *Cbs* ^+/−^ mice and of the infarct area and the remote myocardium at day 28 after ligation of the LAD in control and AdCBS treated C57BL/6 *Ldlr* ^−/−^ *Cbs* ^+/−^ mice.

	Sham	Control MI	AdCBS MI
Capillary density infarct zone (number/mm^2^)	N.A.	143±15	175±13
Collagen deposition infarct zone (% of infarct area)	N.A.	33.7±2.4	42.8±2.1*
Leukocyte count infarct zone (number/mm^2^)	N.A.	1210±160	1180±120
Capillary density remote myocardium (number/mm^2^)	6160±150	3530±390§§§	4420±200§§ *
Cardiomyocyte cross-sectional area (µm^2^)	198±12	252±17§	272±6§§§
Cardiomyocyte density (number/mm^2^)	4770±270	3580±210§§§	3620±90§§§
Relative vascularity (µm^−2^)	0.00669±0.00017	0.00410±0.00043§§§	0.00453±0.00022§§§
Interstitial collagen (%)	2.70±0.41	23.4±1.8§§§	19.1±0.9§§§ *
Perivascular fibrosis (ratio)	0.384±0.011	0.577±0.027§§§	0.538±0.012§§§
Leukocyte count remote myocardium (number/mm^2^)	378±20	373±51	340±271

Data are expressed as means ± S.E.M. (n = 11 for Sham, n = 13 for Control MI, n = 25 for AdCBS MI). N.A.: not applicable.

§ p<0.05;

§§ p<0.01;

§§§ p<0.001 versus Sham.

* p<0.05 versus Control MI.

**Table 6:** Histological parameters of the left ventricle of sham C57BL/6 mice and of sham C57BL/6 *Ldlr* ^−/−^ *Cbs* ^+/−^ and AdCBS treated C57BL/6 *Ldlr* ^−/−^ *Cbs* ^+/−^ mice.

	Sham C57BL/6	Sham C57BL/6*Ldlr* ^−/−^ *Cbs* ^+/−^	Sham AdCBS treatedC57BL/6 *Ldlr* ^−/−^ *Cbs* ^+/−^
Capillary density (number/mm^2^)	6510±120	6160±150	6260±120
Cardiomyocyte cross-sectional area (µm^2^)	202±12	198±12	185±8
Cardiomyocyte density (number/mm^2^)	4630±260	4770±270	4960±190
Relative vascularity (µm^−2^)	0.00721±0.00023	0.00669±0.00017	0.00693±0.00015
Interstitial collagen (%)	1.90±0.20	2.70±0.41	2.85±0.37
Perivascular fibrosis (ratio)	0.288±0.023	0.384±0.011§§§	0.388±0.008
Leukocytes (number/mm^2^)	348±14	378±20	360±15

Sham C57BL/6 mice (n = 10) were fed normal chow. Sham C57BL/6 *Ldlr* ^−/−^ *Cbs* ^+/−^ (n = 11) and AdCBS treated (n = 11) C57BL/6 *Ldlr* ^−/−^ *Cbs* ^+/−^ mice were fed with folate-depleted, methionine-enriched diet supplemented with 0.2% cholesterol and 10% coconut oil. Data are expressed as means ± S.E.M.

§§§ p<0.001 versus C57BL/6.

### Selective Homocysteine Lowering Results in Beneficial Effects on EPC Function

**Figure 6:**
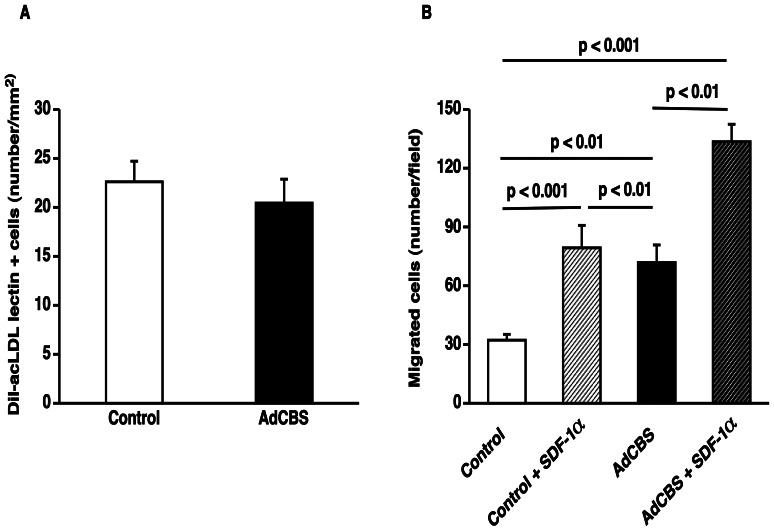
Selective homocysteine lowering gene transfer enhances EPC function. (**A**) Bar graph showing the number of Dil-acLDL FITC-isolectin double positive cells after 7 days of *ex vivo* culture of spleen mononuclear cells isolated at day 14 after Adnull transfer or AdCBS transfer in C57BL/6 *Ldlr* ^−/−^ *Cbs* ^+/−^ mice on a hyperhomocysteinemic and high saturated fat/high cholesterol diet (n = 7 for each group). (**B**) Bar graph showing the number of migrated EPCs in modified Boyden chambers. After 7 days of culture, spleen EPCs isolated at day 14 from saline injected or AdCBS treated C57BL/6 *Ldlr* ^−/−^ *Cbs* ^+/−^ mice were seeded in the upper chamber. In selected experiments, SDF-1α (100 ng/ml) was added in the lower chamber. The number of migrated cells per microscopy field was quantified after 5 hours (n = 12 for each group). Data are expressed as means ± S.E.M.

The beneficial effects of AdCBS gene transfer on capillary density in the remote myocardium and in the infarct zone may be related to increased EPC number and/or EPC function. Therefore, we investigated the effect of AdCBS gene transfer on EPC number and *ex vivo* EPC function. EPCs were isolated from spleens of control and AdCBS mice at day 14 after Adnull or AdCBS gene transfer. After culture for 7 days, EPC number determined as the number of Dil-acLDL FITC-isolectin double positive cells, was not significantly different between both groups (Figure 6A). To evaluate the effect of AdCBS gene transfer on EPC function, EPC migration was analysed (Figure 6B). The number of migrated cells was 2.2-fold (p<0.01) higher for EPCs isolated from AdCBS mice compared to EPCs isolated from control mice. In the presence of 200 ng/ml of SDF-1α, EPC migration was 68% (p<0.01) higher for cells isolated from AdCBS mice compared to cells isolated from control mice (Figure 6B). Therefore, enhanced EPC function may contribute to the observed effects on CD31 positive capillaries.

### Selective Homocysteine Lowering Significantly Improves Diastolic Function after Myocardial Infarction

Hemodynamic parameters in control MI and in AdCBS MI mice at day 28 after ligation of the left anterior descending coronary artery in comparison with data in C57BL/6 *Ldlr* ^−/−^ *Cbs* ^+/−^ mice are shown in Table 7. Diastolic function in AdCBS MI mice was improved compared to control MI mice as evidenced by a 19% (p<0.05) increase of the peak rate of isovolumetric relaxation and a 21% (p<0.05) reduction of the time constant of left ventricular relaxation. The end-diastolic pressure was significantly (p<0.05) lower in AdCBS MI mice compared to control MI mice (Table 7). The peak rate of isovolumetric contraction was non-significantly higher in AdCBS MI mice than in control MI mice. No statistically significant differences were observed in hemodynamic parameters of the three sham groups (Table 8). However, there was a trend for a higher time constant of left ventricular relaxation in the sham C57BL/6 *Ldlr* ^−/−^ *Cbs* ^+/−^ mice and sham AdCBS treated C57BL/6 *Ldlr* ^−/−^ *Cbs* ^+/−^ mice compared to chow fed-C57BL/6 mice (Table 8).

**Table 7:** Hemodynamic parameters in the left ventricle and in the aorta of Sham C57BL/6 *Ldlr* ^−/−^ *Cbs* ^+/−^ mice and *at* day 28 after myocardial infarction in control and AdCBS treated C57BL/6 *Ldlr* ^−/−^ *Cbs* ^+/−^ mice.

	Sham	Control MI	AdCBS MI
**LEFT VENTRICLE**			
Peak systolic pressure(mm Hg)	102±2	94.2±4.3	94.3±3.2
End-diastolic pressure(mm Hg)	0.530±0.413	10.6±2.3§§§	3.50±0.88§ *
dP/dt max (mm Hg/ms)	10.3±0.5	7.62±0.99§	8.63±0.49
dP/dt min (mm Hg/ms)	−9.58±0.35	−5.93±0.41§§§	−7.03±0.27§§§ *
Tau (ms)	4.89±0.28	7.72±0.49§§§	6.12±0.16§ *
Heart rate (bpm)	626±16	613±27	597±12
**AORTA**			
Mean pressure (mm Hg)	85.0±3.2	76.0±3.5	79.1±2.8
Systolic pressure (mm Hg)	100±3	92.1±3.0	93.2±3.0
Diastolic pressure (mm Hg)	70.8±3.1	64.4±3.9	67.4±2.6

Data are expressed as means ± S.E.M.

§ p<0.05;

§§§ p<0.001 versus Sham.

* p<0.05 versus Control MI.

**Table 8:** Hemodynamic parameters in the left ventricle and in the aorta of sham C57BL/6 mice and of sham C57BL/6 *Ldlr* ^−/−^ *Cbs* ^+/−^ and sham AdCBS treated C57BL/6 *Ldlr* ^−/−^ *Cbs* ^+/−^ mice.

	Sham C57BL/6	Sham C57BL/6*Ldlr* ^−/−^ *Cbs* ^+/−^	Sham AdCBS treated C57BL/6 *Ldlr* ^−/−^ *Cbs* ^+/−^
**LEFT VENTRICLE**			
Peak systolic pressure (mm Hg)	98.5±2.9	102±2	98.4±3.0
End-diastolic pressure (mm Hg)	−0.085±0.651	0.530±0.413	0.468±0.605
dP/dt max (mm Hg/ms)	11.3±0.9	10.3±0.5	10.4±1.1
dP/dt min (mm Hg/ms)	−9.73±0.46	−9.58±0.35	−9.39±0.59
Tau (ms)	4.32±0.20	4.89±0.28	4.99±0.32
Heart rate (bpm)	614±26	626±16	593±13
**AORTA**			
Mean pressure (mm Hg)	83.3±2.8	85.0±3.2	81.1±5.0
Systolic pressure (mm Hg)	98.5±2.9	100±3	98.2±4.7
Diastolic pressure (mm Hg)	68.4±3.0	70.8±3.1	65.7±5.1

Sham C57BL/6 mice (n = 10) were fed normal chow. Sham C57BL/6 *Ldlr* ^−/−^ *Cbs* ^+/−^ (n = 11) and AdCBS treated (n = 11) C57BL/6 *Ldlr* ^−/−^ *Cbs* ^+/−^ mice were fed with folate-depleted, methionine-enriched diet supplemented with 0.2% cholesterol and 10% coconut oil. Data are expressed as means ± S.E.M.

## Discussion

The main findings of the present study are that 1) selective homocysteine lowering gene transfer improves infarct healing as evidenced by a shorter infarct length and a higher collagen content and higher number of capillaries in the infarct zone; 2) improved infarct healing in AdCBS MI mice was associated with beneficial effects on late remodelling as indicated by a smaller left ventricular cavity area, a lower interstitial collagen content, and a higher capillary density in the remote myocardium; and 3) histological and structural differences between AdCBS MI mice and control MI mice resulted in a significantly improved diastolic function and a lower end-diastolic pressure in the latter. Taken together, selective homocysteine lowering gene transfer potently attenuated adverse left ventricular remodelling after MI.

Left ventricular remodelling after ligation of the left anterior descending coronary artery is sex dependent with a more pronounced degree of infarct expansion and a higher incidence of ventricular rupture in male mice [27], [28]. To attenuate experimental variability, the study design was restricted to one sex and all experiments were performed in female mice. An important strength of the current study is that homocysteine lowering was induced by selective homocysteine lowering gene transfer. Therefore, dietary effects unrelated to homocysteine lowering cannot have an impact on the end-points in our study. Furthermore, the lipoprotein distribution in the control MI and intervention MI group is significantly more close to human lipoprotein levels compared to wild-type mice. The pertinence of this parameter is highlighted by prior experimental observations showing that a more human-like lipoprotein profile significantly affects cardiac remodelling and cardiac function after MI in mice [14].

Oxidative stress induced by hyperhomcysteinemia enhances nitric oxide inactivation via increased peroxynitrite production as evidenced by elevated nitrotyrosine levels in the myocardium [29]. Nitric oxide and its downstream target, protein kinase G, are generally considered to blunt hypertrophy [30], [31] whereas nitric oxide synthase-3 uncoupling induces marked cardiac hypertrophy, dilation, and dysfunction [32]. Therefore, reduced bioavailability of nitric oxide and hyperhomocysteinemia-induced uncoupling of eNOS [33] may contribute to pathological remodelling.

Fibrosis was increased in the infarct zone following homocysteine lowering, which is consistent with improved infarct healing. Improved infarct healing was also evidenced by an increase of the capillary density in the infarct zone, which may be related to enhanced EPC function. Detrimental effects of hyperhomocysteinemia on neovascularization have previously been demonstrated in the hindlimb ischemia model [34], [35]. The enhanced infarct healing and the consequent reduced infarct length at day 28 may be a primary cause of reduced left ventricular enlargement following homocysteine lowering.

Selective homocysteine lowering gene transfer significantly improved diastolic function post-MI. Diastolic dysfunction may involve the process of active relaxation and/or may reflect abnormalities of passive stiffness. Improved isovolumetric relaxation in AdCBS MI mice compared to control MI mice is reflected by an increased peak instantaneous rate of LV pressure decline and by a decrease of the time constant of isovolumetric relaxation. τ is the time that it takes for LV pressure to fall by approximately two thirds of its initial value. In addition to active relaxation, passive viscoelastic properties contribute to a return of the myocardium to its resting force and length. Although we did not investigate the relationship between diastolic pressure and volume, chamber stiffness may be decreased in AdCBS MI mice as a consequence of the lower collagen content in these mice compared to control MI mice. Myocardial fibrosis has previously been shown to be associated with diastolic dysfunction in hyperhomocysteinemic rats [8], [11]. The attenuated diastolic dysfunction in AdCBS MI mice may have significantly contributed to the lower end-diastolic pressure and reduced pulmonary congestion compared to control MI mice.

Homocysteine lowering not only reduced interstitial fibrosis but also significantly increased capillary density in the remote myocardium. In pathological hypertrophy, mismatch between cardiomyocyte size and vascularity may induce myocardial hypoxia and cardiomyocyte death, which may accelerate progression to congestive heart failure [36]. Therefore, microvascular rarefaction under conditions of hyperhomocysteinemia may have contributed to the development of heart failure in the control MI mice. Overt heart failure in control MI mice was reflected by the increased end-diastolic pressure and the increased lung weight, suggesting pulmonary congestion.

This experimental animal intervention study should be seen in light of epidemiological studies that suggest a potential contribution of elevated homocysteine levels to heart failure development in humans. Firstly, plasma homocysteine levels were directly related to left ventricular mass and wall thickness in women but not in men in the Framingham Heart Study participants that were free of heart failure and previous myocardial infarction [1]. Secondly, elevated homocysteine levels were associated with reduced regional left ventricular systolic function determined by tagged magnetic resonance imaging in asymptomatic subjects [2]. Thirdly, plasma homocysteine levels correlate with clinical, echocardiographic, and laboratory parameters of the severity of heart failure [4]. Fourthly, elevated plasma homocysteine was associated with an increased incidence of congestive heart failure in the Framingham Heart Study [3]. This association remained significant in multivariable analyses controlling for established risk factors for congestive heart failure including the occurrence of myocardial infarction during follow-up [3]. The current experimental animal intervention study supports the hypothesis that epidemiological associations may reflect a causal relationship.

A limitation of the current study is that the homocysteine levels in the control MI group (approximately 95 µM) were higher than in humans with mild hyperhomocysteinemia (15–30 µM) and most individuals with moderate hyperhomocysteinemia (31–100 µM). A second limitation is that the duration of follow-up was restricted to 28 days. In addition, gene transfer in the current study was performed two weeks before MI and not after MI to exclude detrimental effects of innate immune responses occurring in the first 24 hours after gene transfer with E1E3E4-deleted adenoviral vectors [37]. This limitation may be overcome by use of adeno-associated viral vectors, which result in very minor innate immune responses [38]. Finally, experiments were performed in a model of combined hypercholesterolemia and hyperhomocysteinemia. The question whether beneficial effects of selective homocysteine lowering gene transfer would also be observed in normocholesterolemic *Cbs^+/−^* mice cannot be answered at present.

In conclusion, improved infarct healing and attenuated adverse remodelling following selective homocysteine lowering gene transfer significantly enhance diastolic function after MI in female C57BL/6 *Ldlr* ^−/−^ *Cbs* ^+/−^ mice. These data corroborate the view that hyperhomocysteinemia exerts direct cardiac effects and may potentiate the development of heart failure.
